# Unlocking the Potential of Auricular Therapy: A Promising Solution for Lumbar Disc Herniation Pain

**DOI:** 10.1155/prm/5722429

**Published:** 2026-09-09

**Authors:** Hui-juan Li, Ting Wang, An-li Huang, Juan Yuan

**Affiliations:** ^1^ School of Nursing, Anhui University of Chinese Medicine, Hefei, China, ahtcm.edu.cn; ^2^ School of Nursing, Anqing Medical College, Anqing, China; ^3^ Key Laboratory of Geriatric Nursing and Health, Anhui University of Chinese Medicine, Hefei, China, ahtcm.edu.cn; ^4^ Nursing Department, The Second Affiliated Hospital of Anhui University of Chinese Medicine, Hefei, China

**Keywords:** auricular acupuncture, auricular pressure point, auricular therapy, lumbar disc herniation, systematic evaluation, traditional Chinese medicine care

## Abstract

**Background:**

Lumbar disc herniation (LDH) is a common degenerative spinal disorder causing low back pain and radiating pain in lower extremities, significantly impairing quality of life. Auricular therapy, a non‐pharmacological approach in Chinese medicine, shows promise for pain management. However, current studies vary in design quality, with limited evidence‐based support.

**Objective:**

To assess the clinical efficacy and safety of auricular therapy for LDH‐related pain through systematic evaluation, providing evidence‐based guidance for clinical management.

**Methods:**

A comprehensive search of major databases (China National Knowledge Infrastructure [CNKI], VIP, SinoMed, Wanfang, PubMed, Web of Science, The Cochrane Library, and Embase) was conducted through December 1, 2025, identifying randomized controlled trials (RCTs) of auricular therapy for LDH. Meta‐analysis was performed using RevMan 5.4 software, with evidence quality assessed using GRADE methodology.

**Results:**

Sixteen RCTs involving 1352 patients were included. The auricular therapy group showed significantly greater improvement compared to controls in Visual Analogue Scale (VAS) scores [MD = −1.05, 95% CI (−1.32, −0.78), (*p* < 0.00001)], Japanese Orthopedic Association (JOA) scores [MD = 3.20, 95% CI (2.16, 4.24), (*p* < 0.00001)], clinical efficacy rate [RR = 1.12, 95% CI (1.07, 1.18), (*p* < 0.00001)], and lower incidence of adverse events [RR = 0.27, 95% CI (0.09, 0.77), (*p* = 0.01)]. The GRADE assessment showed moderate‐quality evidence for clinical efficiency, low‐quality evidence for JOA scores and adverse events, and very low‐quality evidence for VAS scores.

**Limitations:**

Most included studies had inadequate randomization procedures and lacked allocation concealment. None included long‐term follow‐up data. All included studies were published in Chinese, limiting global generalizability and potentially missing high‐quality international research.

**Conclusions:**

Current evidence suggests auricular therapy effectively alleviates pain in LDH patients and enhances treatment efficacy. However, due to methodological limitations and low evidence quality for some outcomes, rigorous multicenter RCTs with larger samples are needed to validate these findings.

## 1. Introduction

Lumbar disc herniation (LDH) is a prevalent degenerative spinal disorder that affects 2%–4% of the global population, causing lower back pain and radiating pain in the lower extremities. Severe cases may progress to cauda equina syndrome, leading to significant disability. The condition is increasingly affecting younger populations due to lifestyle changes [1–3]. Pain, one of the earliest and most common symptoms of LDH, significantly impairs patients’ daily activities, work capacity, and quality of life while often resulting in limited mobility [4, 5].

Non‐surgical treatments are preferred for non‐acute cases, but pharmacological options often have adverse effects with prolonged use [6]. In recent years, traditional Chinese medicine (TCM) has gained recognition as an adjunctive treatment for LDH pain due to its simplicity, affordability, safety, and minimal side effects [7].

Ear acupuncture therapy (auricular therapy) is a method of preventing and treating diseases by stimulating corresponding reflex zones on the ear through techniques such as auricular pressure point stimulation, auricular acupuncture, and auricular massage. Research suggests that ear acupuncture therapy holds potential for pain relief [8]. Despite its potential, the clinical efficacy of auricular therapy for LDH lacks robust evidence‐based support. This study systematically reviews and analyzes existing randomized controlled trials (RCTs) to evaluate the efficacy and safety of auricular therapy for LDH‐related pain, providing guidance for clinical practice.

## 2. Literature and Methods

This study followed the PRISMA (Preferred Reporting Items for Systematic Reviews and Meta‐Analyses) checklist [9], and the protocol was registered with PROSPERO (ID number: CRD42025626592).

### 2.1. Inclusion and Exclusion Criteria

The inclusion criteria for this review were as follows: (1) the included studies were RCTs; (2) the study subjects had to be patients diagnosed with LDH who reported low back pain or radiating pain in the lower extremities, with clearly defined inclusion and exclusion criteria. There were no restrictions on age, gender, disease duration, or severity; (3) the control group received non‐auricular point therapy, while the experimental group received auricular point therapy either as a standalone intervention or in combination with the control group’s treatment (including auricular pressure point and auricular acupuncture); (4) the studies reported at least one of these outcome measures or provided data that can be used to calculate them, including Visual Analogue Scale (VAS) scores, Japanese Orthopedic Association (JOA) scores, clinical efficacy rates, and adverse reactions.

The exclusion criteria were as follows: (1) non‐RCT literature such as review articles, animal experiments, and case reports; (2) studies in which the full text or complete data weren’t available; and (3) RCTs with multiple control groups.

### 2.2. Literature Search Strategy

The Chinese electronic databases that we searched included China National Knowledge Infrastructure (CNKI), Chinese Scientific Journal Database (VIP), SinoMed, and Wanfang. The English electronic databases included PubMed, Web of Science, The Cochrane Library, and Embase. The search period for all databases spanned from their inception to December 1, 2025. Taking PubMed as an example, the search formula was as follows:

 1#Auricular Point OR Auricular Acupressure OR Ear Acupressure OR Acupuncture, Ear
 2#(((((((Intervertebral Disc Displacement [MeSH Terms]) OR (Disc Displacement, Intervertebral [Title/Abstract])) OR (Intervertebral Disc Displacements [Title/Abstract])) OR (Protruded Disc [Title/Abstract])) OR (Disc, Protruded [Title/Abstract])) OR (Discs, Protruded [Title/Abstract])) OR (Slipped Disk [Title/Abstract])) OR (Disk, Slipped [Title/Abstract])
 3#1#AND2#

### 2.3. Literature Screening and Data Extraction

Two researchers independently screened the literature and extracted data. Disagreements were resolved through discussion or consultation with a third researcher. All the included RCTs were approved by the ethics committee, and the informed consent forms were signed by the participants. The extracted information included author details, sample size, treatment duration, intervention methods, characteristics of auricular therapy, and outcome measures.

### 2.4. Literature Quality Evaluation

The quality of the included studies was assessed using the Cochrane Collaboration’s Risk of Bias tool [10]. The evaluation criteria included random sequence generation, allocation concealment, blinding implementation, outcome data completeness, selective reporting, and other potential sources of measurement bias. Each study was classified as having a low risk, unclear risk, or high risk of bias across these domains.

### 2.5. Statistical Analysis of Data

The meta‐analysis was performed using RevMan 5.4.1 software. (1) Regarding the effect size selection for binary outcomes, the relative risk (RR) with a 95% confidence interval (CI) was selected. For continuous outcomes, the weighted mean difference (MD) or standardized mean difference (SMD) with a 95% CI was selected. (2) For heterogeneity assessment of the meta‐analysis, a *χ* ^2^ test was used to assess heterogeneity. If studies exhibited homogeneity (*p* > 0.1, *I* ^2^ < 50%), a fixed‐effect model was applied; otherwise, a random‐effects model was adopted. Heterogeneity was addressed through subgroup analysis or sensitivity analysis via sequential deletion. The significance level was set at *α* = 0.05.

### 2.6. Grading the Certainty of Evidence for Major Comparisons and Outcomes

The GRADE approach [11] was used to evaluate evidence quality across five domains: risk of bias, inconsistency, indirectness, imprecision, and reporting bias. Two reviewers independently assessed each domain for all outcomes.

## 3. Results

### 3.1. Literature Retrieval and Screening

**FIGURE 1:**
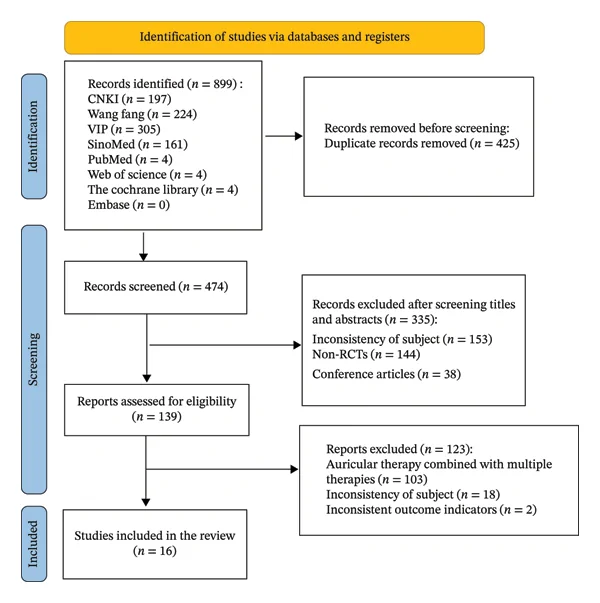
Flowchart of the literature selection process.

A total of 899 articles were retrieved from the database, including 887 Chinese‐language articles and 12 English‐language articles. After removing duplicates, 474 articles remained. These articles were further screened based on the predefined inclusion and exclusion criteria, ultimately resulting in the final inclusion of 16 articles, as shown in Figure 1.

### 3.2. Basic Characteristics of the Included Studies

The included studies were published between 2011 and 2025, involving a total of 1352 patients. Among them, 675 patients were in the auricular therapy group, including 401 patients in the auricular pressure point group and 274 in the auricular acupuncture group. The remaining 677 patients were in the control group. Detailed information on the included studies is provided in Table 1.

**TABLE 1:** Basic information of the included literature.

Author	Sample size (experimental group/control group)	Course of treatment	Methods of intervention		Auricular therapy			Outcome measures
			Trial groups	Control group	Number of compressions	Each press time	Selection of acupoints	
Ye 2018 [12]	30/30	3 days	Auricular pressure point + analgesic pump	Intravenous analgesia pump	3 times per day	1 min	Shenmen, subcortical, lumbar vertebrae, tri‐jiao, concha auriculata	^1^, ^4^
Cao 2024 [13]	32/31	28 days	Comprehensive auricular therapy + Traditional Chinese medicine fumigation and washing	Traditional Chinese medicine fumigation and washing	3 times per day	50 compressions each time	Lumbar triangle, liver, kidney, Shenmen, Sanjiao	^1^, ^3^
Lin 2022 [14]	40/40	1 month	Auricular pressure point + Lumbar traction, Local hot compress, Electromagnetic acupuncture	Lumbar traction, local hot compress, electromagnetic acupuncture	5 times per day	10 compressions each time	Shenmen, subcortical, sympathetic	^1^
Li 2014 [15]	30/30	4 weeks	Auricular acupuncture + Electroacupuncture, cupping, TDP	Electroacupuncture, cupping, TDP	1 time per day	30 min	Shenmen, lumbosacral vertebrae, sciatic nerve	^1^, ^2^, ^3^
Liu 2021 [16]	33/32	4 weeks	Auricular acupuncture + Jiaji warming acupuncture therapy	Jiaji warming acupuncture therapy	1 time per day	30 min	Shenmen, subcortical, liver, kidney, lumbosacral vertebrae, sciatic nerve	^1^, ^2^, ^3^
Chen 2011 [17]	48/48	1 week	Auricular pressure point + Jinbei decoction orally	In decoction orally	3 times per day	5 min	Lumbosacral vertebrae, Shenmen, sciatic nerve	^1^, ^4^
Ye 2019 [18]	37/37	5 days	Auricular pressure point + Usual care	Usual care	3 times per day	3–5 min	Shenmen, tri‐jiao, subcortical, lumbosacral vertebrae	^1^
Meng 2023 [19]	34/33	20 days	Auricular pressure point + Electroacupuncture	electroacupuncture	3 times per day	30 compressions each time	Lumbosacral vertebrae, gluteal, sciatic nerve, Shenmen, subcortical, endocrine, kidney, liver	^1^, ^2^, ^3^
Xu 2019 [20]	48/48	2 weeks	Auricular pressure point + Jinbei decoction orally	Jinbei decoction orally	3 times per day	5 min	Lumbosacral vertebrae, Shenmen, sciatic nerve	^1^, ^4^
Zhang 2021 [21]	42/45	5 days	Auricular pressure point + Usual care	Usual care	4 times per day	30 compressions each time	Shenmen, San Jiao, subcortical, lumbosacral vertebrae, liver, kidney	^1^
Yu 2023 [22]	49/49	3 days	Auricular acupuncture + Local infiltration analgesia	Local infiltration analgesia	3–4 times per day	1 min	Shenmen, sympathetic, subcortical	^4^
Yang 2020 [23]	32/34	4 weeks	Auricular acupuncture + Electroacupuncture	electroacupuncture	3 times per day	10–20 compressions each time	Lumbosacral vertebrae, Shenmen, sympathetic, kidney	^1^, ^2^, ^3^
Fan 2016 [24]	30/30	3 weeks	Auricular acupuncture + Electroacupuncture	electroacupuncture	3 times per day	10 compressions each time	The sciatic nerve, Shenmen, lumbosacral vertebrae, buttock, kidney, liver	^1^, ^2^, ^3^
Xiong 2024 [25]	100/100	1 week	Auricular acupuncture + Electroacupuncture, cupping therapy, and other treatments	Electroacupuncture, cupping therapy, and other treatments	3 times per day	10 compressions each time	Shenmen, kidney, lumbosacral vertebrae, sciatic nerve	^1^, ^2^, ^3^
Yang 2017 [26]	40/40	1 week	Auricular pressure point + TCM Herbal fomentation	TCM Herbal fomentation	3 times per day	30–50 compressions each time	Waist, kidney, liver, Shenmen	^1^, ^3^
Liu 2023 [27]	50/50	5 days	Auricular pressure point + Usual care	Usual care	4–5 times per day	3 min	Lumbosacral vertebrae, Shenmen, kidney, subcortical	^1^, ^2^, ^3^

^1^VAS score.

^2^JOA score.

^3^Clinical efficacy rate.

^4^Adverse reactions.

### 3.3. Quality Evaluation of the Included Literature

**FIGURE 2:**
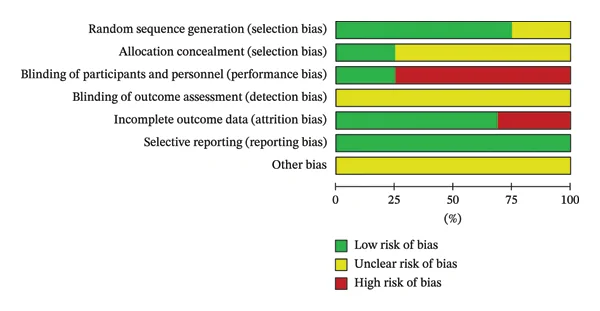
Proportion of bias risk graph.

**FIGURE 3:**
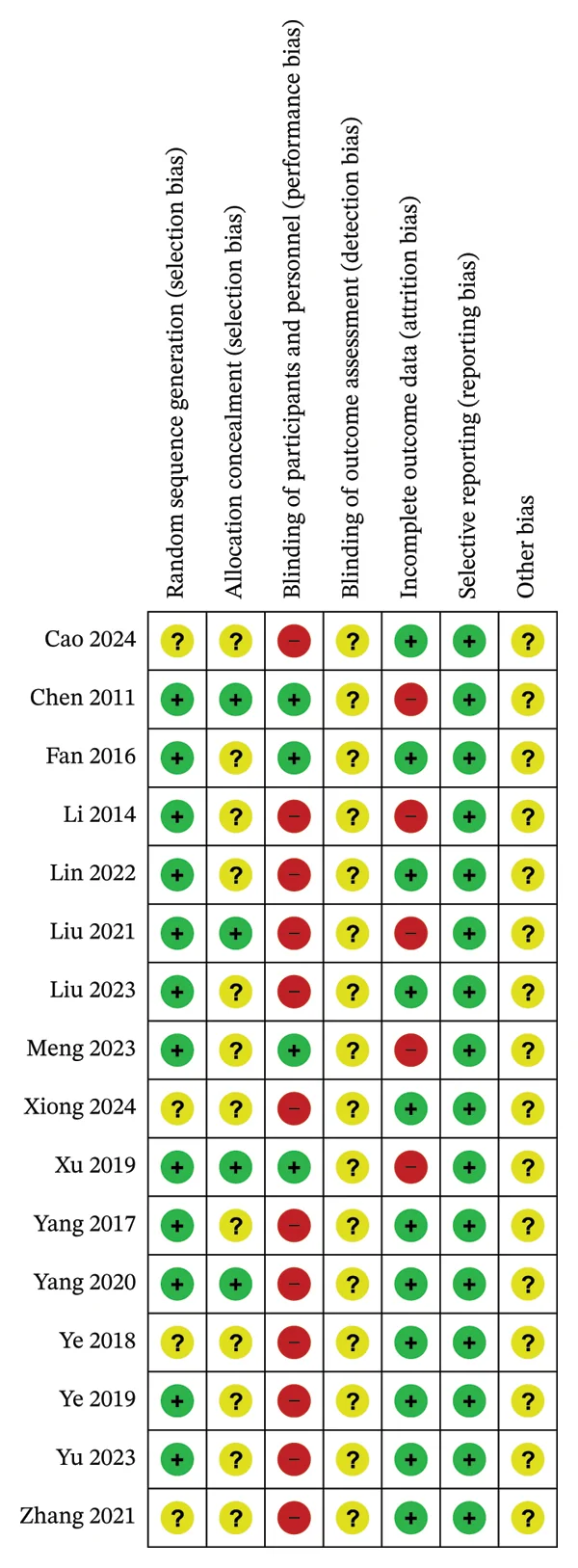
Summary of bias risk outcome measurements.

The Cochrane risk of bias assessment tool was used to evaluate the risk of bias in the included studies. Among the 16 included studies, four studies [12, 13, 21, 25] did not specify the grouping method and were therefore evaluated as “unclear.” The remaining 12 studies used the random number table method, which was evaluated as “low risk.” Four studies [16, 17, 20, 23] reported random sequence allocation concealment and therefore were evaluated as “low risk,” while the rest did not report it and were evaluated as “unclear.” Four studies [17, 19, 20, 24] adopted the patient and interventionist blinding method. Among them, two studies [19, 24] did not disclose the relevant information about the group treatment to the patients, and the treatment operators, efficacy evaluators, and statistical analysts were separated to ensure blinding. Two studies [17, 20] achieved blinding through the use of sham auricular point therapy. This was evaluated as “low risk.” The rest of the studies did not mention blinding; however, some studies mentioned that due to the inherent nature of TCM external treatment techniques, it is impossible to achieve blinding for both patients and interventionists, so they were evaluated as “high risk.” Five studies [15–17, 19, 20] reported a loss to follow‐up, so the integrity of their data was evaluated as “high risk.” The rest of the studies did not mention any loss to follow‐up, but since the number of recruited participants matched the number included in the analysis, they demonstrated good data integrity and were evaluated as “low risk.” All studies reported the results of all observed indicators, so the reporting bias was evaluated as “low risk.” However, none of the 16 included studies mentioned the registration situation; thus, other biases were evaluated as “unclear.” The detailed assessment results are presented in Figures 2 and 3.

### 3.4. Results

#### 3.4.1. VAS Score

**FIGURE 4:**
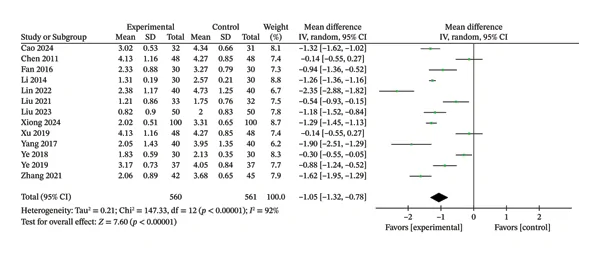
The meta‐analysis results comparing VAS scores between the two groups.

Thirteen studies [12–18, 20, 21, 24–27] reported the effect of auricular acupressure on VAS scores, including a total of 1121 patients (560 in the experimental group and 561 in the control group), where higher VAS scores indicate greater pain severity. Meta‐analysis of the results demonstrated a statistically significant difference between the auricular acupressure group and the control group in terms of VAS scores [MD = −1.05, 95% CI (−1.32, −0.78), (*p* < 0.00001)], indicating that the auricular therapy group was superior to the control group, as shown in Figure 4.

Due to the high heterogeneity of the results (*p* < 0.00001, I^2^ = 92%), subgroup analyses of VAS scores were conducted based on patient age, intervention method, and the course of the intervention. The subgroup analyses by age showed that LDH patients younger than 40 years old had significantly lower VAS scores [MD = −2.35, 95% CI (−2.88, −1.82), (*p* < 0.00001)]. These findings suggest that age differences may be the source of high heterogeneity in this study. However, since only a limited number of studies included this factor, the results should be interpreted with caution. The detailed findings are presented in Table 2.

**TABLE 2:** Subgroup Analysis of VAS scores.

Subgroup	Subgroup	Number of papers	*n*	MD	95% CI	*Z*	*p*
Age	< 40 years	1	80	−2.35	[−2.88, −1.82]	8.68	< 0.00001
	≥ 40 years	12	1041	−0.95	[−1.22, −0.69]	7.08	< 0.00001

Intervention course	> 2 weeks	6	424	−1.08	[−1.51, −0.65]	4.95	< 0.00001
	≤ 2 weeks	7	697	−1.03	[−1.46, −0.60]	4.68	< 0.00001

Intervention	Ear acupuncture + Electroacupuncture	3	320	−1.25	[−1.35, −1.15]	24.90	< 0.00001
	Auricular pressure point + Usual care	3	261	−1.23	[−1.65, −0.81]	5.72	< 0.00001
	Auricular pressure point + Jinbei decoction orally	2	192	−0.14	[−0.43, 0.15]	0.95	0.34
	Auricular pressure point + Other therapies	5	348	−1.25	[−1.97, −0.54]	3.45	0.0006

#### 3.4.2. JOA Score

**FIGURE 5:**
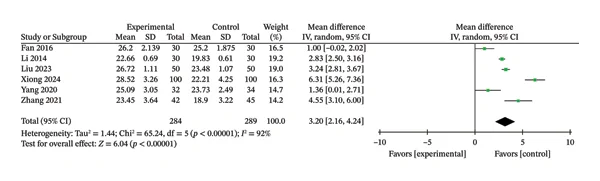
The meta‐analysis results comparing JOA scores of the two groups.

Six studies [15, 21, 23–25, 27] reported JOA scores, including a total of 573 patients (284 in the experimental group and 289 in the control group). Higher JOA scores indicated better recovery of low back function. Analysis of the results showed a statistically significant difference between the auricular acupuncture therapy group and the control group in terms of JOA scores [MD = 3.20, 95% CI (2.16, 4.24), (*p* < 0.00001)], indicating that the auricular acupuncture therapy group was superior to the control group. The detailed results are presented in Figure 5.

After conducting a heterogeneity test, the results showed substantial heterogeneity between studies (*p* < 0.00001, *I* ^2^ = 92%). Subgroup analyses of JOA scores were then conducted using the intervention course and intervention method. The analysis by intervention course revealed that patients who underwent auricular therapy for ≤ 2 weeks had significantly higher JOA scores [MD = 4.67, 95% CI (2.60, 6.74), (*p* < 0.00001)]. In the subgroup of patients with an intervention course of more than 2 weeks, JOA scores were still higher in the experimental group compared to the control group [MD = 1.81, 95% CI (0.45, 3.18), (*p* = 0.009)]. These findings suggest that the cause of the high study heterogeneity may be related to the intervention regimen. The detailed results are presented in Table 3.

**TABLE 3:** Subgroup analysis of JOA scores.

Subgroup	Subgroup	Number of papers	*N*	MD	95% CI	*Z*	*p*
Intervention course	> 2 weeks	3	186	1.81	[0.45, 3.18]	2.61	0.009
	≤ 2 weeks	3	387	4.67	[2.60, 6.74]	4.42	< 0.00001

Intervention method	Ear acupuncture + Electroacupuncture	4	386	2.89	[0.99, 4.79]	2.98	0.003
	Auricular pressure point + Usual care	2	187	3.70	[2.48, 4.93]	5.91	< 0.00001

#### 3.4.3. Clinical Efficacy

**FIGURE 6:**
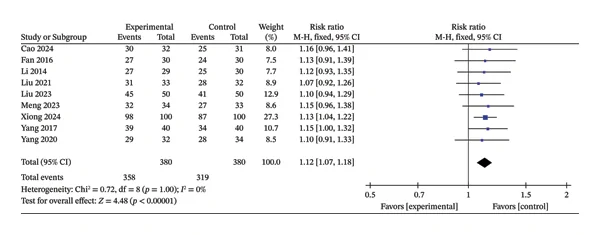
The meta‐analysis results comparing clinical efficacy between the two groups.

Nine studies [13, 15, 16, 19, 23–27] compared the clinical efficacy of auricular therapy versus non‐auricular therapy, including a total of 760 patients (380 cases in the experimental group and 380 in the control group). After conducting a heterogeneity test, the results showed low heterogeneity between studies (*p* = 1.00, *I* ^2^ = 0%), so a fixed‐effect model was used. The meta‐analysis results showed that auricular therapy significantly improved the clinical efficacy of LDH patients [RR = 1.12, 95% CI (1.07, 1.18), (*p* < 0.00001)]. The detailed results are presented in Figure 6.

#### 3.4.4. Incidence of Adverse Reactions

**FIGURE 7:**
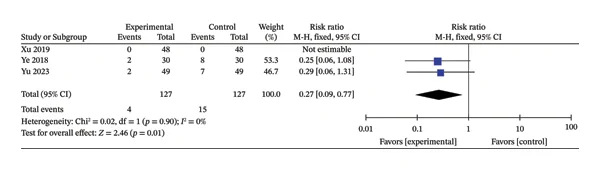
The meta‐analysis results comparing the incidence of adverse reactions between the two groups.

Three studies [12, 20, 22] reported adverse reactions, including a total of 257 patients (127 in the experimental group and 127 in the control group). The heterogeneity test indicated low heterogeneity among the studies (*p* = 0.90, *I* ^2^ = 0%), so a fixed‐effect model was adopted. The meta‐analysis results showed that the incidence of adverse reactions in the auricular therapy group was significantly lower than that in the control group, with a statistically significant difference [RR = 0.27, 95% CI (0.09, 0.77), *p* = 0.01], as shown in Figure 7.

#### 3.4.5. Publication Bias

**FIGURE 8:**
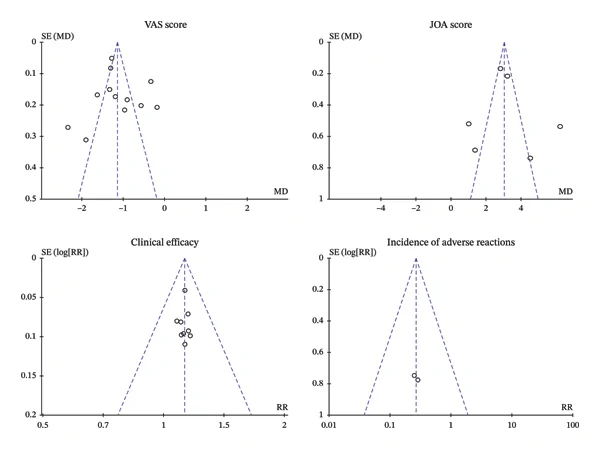
Funnel plot of auricular therapy intervention for LDH pain outcome indicator.

The publication bias analysis of each outcome indicator showed that the bilateral distribution of VAS scores was asymmetric, suggesting significant bias. In contrast, JOA scores, clinical efficacy rate, and incidence of adverse reactions were symmetric, with no significant bias, as shown in Figure 8. However, given that all the studies included in this meta‐analysis were published literature, the possibility of unpublished studies with negative results cannot be ruled out. Therefore, we remained cautious regarding potential publication bias in the included literature.

#### 3.4.6. GRADE Evidence Rating

A GRADE assessment was conducted for each outcome indicator. Some of the included studies lacked descriptions of protocol concealment and blinding, leading to a serious risk of bias. There was heterogeneity in both the VAS and JOA scores between the outcome indicators, so the inconsistency of these two indicators was rated as “serious.” Since the outcome indicators were direct evidence, the indirectness was considered insignificant. Only three papers reported adverse reactions, which lowered the precision of the incidence of adverse reactions, so it was rated as “serious.” Among the outcome indicators, VAS scores demonstrated significant bias, which was rated as “serious.” However, no significant bias was found in the other indicators, so the publication bias was rated as “not detected.” In summary, the VAS score was classified as very low‐quality evidence, the JOA score and the incidence of adverse effects were classified as low‐quality evidence, and the clinical efficacy rate was classified as moderate‐quality evidence, as shown in Table 4.

**TABLE 4:** GRADE evidence grading of the included literature.

Outcome indicator	Evaluation of the quality of evidence					Sample size (incidence)		Effect size (95% CI)	Quality grading (GRADE)
	Risk of bias	Inconsistency	Indirectness	Imprecision	Reporting bias	Experimental group	Control group		
VAS score	Serious	Serious	Insignificant	Insignificant	Serious	560	561	MD = −1.13 [‐1.20, −1.06]	Very low
JOA score	Serious	Serious	Insignificant	Insignificant	Not found	284	289	MD = 3.04 [2.80, 3.28]	Low
Clinical efficacy	Serious	Insignificant	Insignificant	Insignificant	Not found	358/380 (94.2%)	319/380 (83.9%)	RR = 1.12 [1.07, 1.18]	Middle
Incidence of adverse reaction	Serious	Insignificant	Insignificant	Serious	Not found	4/127 (3.1%)	15/127 (11.8%)	RR = 0.27 [0.09, 0.77]	Low

## 4. Discussion

### 4.1. Methodological Quality Assessment

This systematic review demonstrated that auricular therapy effectively alleviates pain in LDH patients. However, the methodological quality of included studies was moderate, with key limitations: (1) inadequate reporting of randomization methods and allocation concealment in most studies, and (2) challenges in implementing blinding procedures due to the nature of TCM interventions.

### 4.2. Auricular Therapy Effects on Pain Symptoms in LDH Patients

Our findings show that auricular therapy, whether as a standalone treatment or combined with conventional therapies, significantly improved pain scores, functional outcomes, and overall clinical efficacy while reducing adverse events. These results suggest auricular therapy can serve as an effective adjunctive treatment for LDH pain management, consistent with previous systematic reviews [28, 29].

### 4.3. Mechanism of Auricular Therapy in Alleviating Pain Symptoms

Modern medicine has not yet fully elucidated the specific pathophysiological mechanisms of LDH, but it is believed to be mainly related to mechanical compression, autoimmune reactions of the intervertebral disc, and inflammatory responses affecting the nerve roots. Current research shows that stimulating auricular points can prompt the body to release endogenous opioid peptides, such as *β*‐endorphin. These substances bind to opioid receptors in the nervous system, blocking the transmission of pain signals and producing analgesic effects similar to those of morphine [30]. Stimulating auricular points has also been found to activate the vagus nerve, which inhibits the production of inflammatory cytokines such as interleukin‐1*β* (IL‐1*β*) and tumor necrosis factor‐*α* (TNF‐*α*) by releasing acetylcholine and other neurotransmitters, thereby reducing inflammation and alleviating inflammation‐induced pain [31].

From the perspective of TCM, LDH falls under the category of “bi syndrome,” which is believed to result from the invasion of wind, cold, and dampness, leading to qi stagnation and blood stasis in the lumbar region and obstruction of the meridians [32]. Auricular points serve as key anatomical locations where the auricle connects with the meridians and internal organs, acting as sites for the transmission of pulse energy. Each organ in the body is thought to correspond to a specific reflex area in the ear. Therefore, pressing or needling auricular points and stimulating the corresponding acupoints can effectively treat diseases [33].

In auricular therapy, commonly selected acupoints include the lumbosacral vertebrae, kidney, Shenmen, and subcortical regions. The recommended frequency of auricular acupressure is 3–5 times/d, 1–3 min per press, and the typical length of intervention is 1–2 weeks. The lumbosacral vertebrae acupoints are considered key points for pain relief, allowing qi to reach the affected area and unblock the meridians, promoting blood circulation and relieving pain. The kidney acupoint is associated with relieving soreness and weakness in the waist and knees. The Shenmen acupoint has anti‐inflammatory, pain‐relieving, and mind‐calming effects. The subcortical acupoint plays a role in the excitation and inhibition of the cerebral cortex, correcting autonomic nervous system disorders and relieving cerebral tension while also exhibiting anti‐inflammatory, antispasmodic, and analgesic effects. These acupoints work together through neural and meridian‐based transmissions to exert mind‐calming, anti‐inflammatory, and analgesic effects, and enhance circulation in meridians and muscles, thereby effectively alleviating the symptoms of low back and leg pain associated with LDH.

### 4.4. Safety Assessment of Auricular Therapy

Only three studies reported adverse reactions, suggesting potential underreporting due to small sample sizes, short observation periods, perceived safety of auricular therapy, or methodological limitations in monitoring adverse events. Future studies should implement systematic adverse event monitoring with standardized assessment tools and explicit protocols.

### 4.5. Limitations of the Research Results

Despite the positive findings, several limitations should be acknowledged:

Methodological issues: Most included studies had inadequate randomization procedures, lacked allocation concealment, failed to implement blinding, and provided no clinical registration information. None included long‐term follow‐up data, limiting assessment of lasting effects.

Evidence quality: High heterogeneity was observed for VAS and JOA scores, potentially due to varying intervention protocols, patient characteristics, and treatment durations.

Literature scope: All included studies were published in Chinese, limiting global generalizability and potentially missing high‐quality international research.

Future research should address these limitations through the following: Rigorous adherence to CONSORT guidelines for RCT reporting; multicenter studies with larger sample sizes to improve generalizability; standardized auricular acupoint stimulation protocols; extended follow‐up periods (3–6 months) to assess long‐term outcomes; more objective outcome measures to provide reliable evidence; and international collaboration to validate findings across diverse populations.

## 5. Conclusion

Current evidence suggests that auricular therapy effectively alleviates pain in LDH patients and enhances overall treatment efficacy. However, due to methodological limitations and the low quality of evidence for some outcomes, rigorous multicenter RCTs with larger samples are needed to validate these findings and optimize clinical protocols. Future research should focus on standardizing intervention methods, exploring underlying mechanisms, and assessing long‐term efficacy to strengthen the evidence base for auricular therapy in LDH management.

## Funding

No funding was received for this manuscript.

## Conflicts of Interest

The authors declare no conflicts of interest.

## Data Availability

The data that support the findings of this study are available from the corresponding author upon reasonable request.
